# Determination of Two-Stage Heat Treatment Parameters in Industrial Conditions in Order to Obtain a TRIP Structure in Low-Alloy Carbon Steel Wires

**DOI:** 10.3390/ma15248965

**Published:** 2022-12-15

**Authors:** Sylwia Wiewiórowska, Marek Siemiński, Tomasz Śleboda, Aneta Łukaszek-Sołek, Tomasz Dyl, Bartosz Koczurkiewicz

**Affiliations:** 1 Faculty of Production Engineering and Materials Technology, Czestochowa University of Technology, Av. Armii Krajowej 19, 42-201 Czestochowa, Poland; 2 Metalurgia S.A. Radomsko, St. Św. Rozalii 10/12, 97-500 Radomsko, Poland; 3 Faculty of Metals Engineering and Industrial Computer Science, AGH University of Science and Technology, Av. Mickiewicza 30, 30-059 Krakow, Poland; 4 Department of Marine Maintenance, Faculty of Marine Engineering, Gdynia Maritime University, Morska Street 81-87, 81-225 Gdynia, Poland

**Keywords:** multiphase steel, TRIP steel, heat-treatment process, wires, drawing process

## Abstract

The research presented in this article aimed to obtain a semi-finished product in the form of TRIP wires, which in further research will be used to produce fasteners in the form of KPS-6 screws used in the construction industry. At present, the process of manufacturing this type of fastener (from wire rod to the finished product) involves two technological lines: one for carrying out the drawing process and obtaining a semi-finished product in the form of a wire with appropriate properties, and the other for the production of fasteners. Semi-finished product wires with a ferritic-perlitic structure obtained after the drawing process are the starting product for the production of fasteners, the tensile strength of which is approximately 450 MPa. In order to be able to obtain fasteners characterized by an increased level of properties in 8.8 grade, after the screw manufacturing process, heat treatment should be carried out by hardening and tempering. The new technology proposed in the article includes: a drawing wire rod with a semi-finished product diameter, two-stage heat treatment on the line for pass-through heating and cooling, ensuring the obtaining of a TRIP-type structure in drawn wires, and calibration drawing. The product of this process was a wire whose tensile strength was in the range of 700–800 MPa with a TRIP structure. Thanks to obtaining a TRIP-type structure with the assumed amount of retained austenite, we obtained wires with higher strength properties and very high plasticity in relation to wires with the same chemical composition and ferritic and perlitic structure. The research carried out in the article also allowed us to obtain, in the semi-finished product wires, a favourable relationship between the strength properties and plasticity of the material, expressed by the value of the R_e_/R_m_ coefficient (yield strength/tensile strength) and the so-called yield ratio, which determines the material′s susceptibility to cold deformation; the smaller these coefficients, the greater the yield strength. The subsequent stages of the research will include the development of forming fasteners in the form of KPS-6 screws used in the steel construction industry with TRIP structures, with increased properties of products in the 8.8 property class, without conducting heat treatment by hardening and tempering. It is assumed that the resulting product will have an additional usable feature: preserving a certain amount of retained austenite in the structure of the finished fasteners, which will be transformed into martensite during operation, and thus affect the longevity of the fasteners.

## 1. Introduction

One of the groups of new-generation structural steels is AHSS (Advanced High-Strength Steel) [1,2,3,4,5,6]. This group includes TRIP (Transformation-Induced Plasticity)-type steels. The high mechanical properties (strength and plasticity) of TRIP steel are obtained thanks to the phenomenon of additional plasticity during the transformation of metastable retained austenite into martensite during plastic deformation (Umwandlungsplastizit in Wassermann, 1937 [7], or Transformation-Induced Plasticity (TRIP) in Zackay, 1969 [8]) [9]. The TRIP effect occurs in three types of steel: austenitic stainless steels, nickel steels for cryogenic operation and low-alloy carbon steels with the addition of manganese, aluminium and/or silicon or phosphorus [10,11,12,13,14,15,16,17,18,19]. A TRIP-type structure in low alloy carbon steels can be obtained using a two-stage heat treatment process. The first step of the hotter treatment involves the annealing process of the material at the temperature T1, in the two-phase austenitic–ferritic range (between the temperatures Ac1 and Ac3), at a convenient time (t1). After annealing in the two-phase range, the steel should be cooled to the bainitic transformation temperature range (T2) with the highest possible cooling speed (v1), and the heating process at this temperature should be carried out for a specified time (t2) [20].

The currently developed technologies for obtaining new-generation steel products virtually exclusively concern the rolling and cooling processes of sheet metal, while the available literature does not describe the technology of manufacturing wire rods and multi-phase steel wires with high strength properties [21,22,23]. Sheets of high-strength, modern steel with a TRIP effect can be obtained both in the hot- and cold-rolling processes. In industrial conditions, steel sheets with the TRIP effect are obtained through the cold-rolling process on hot-dip galvanizing lines and through the processes of regulated cooling after the hot-rolling process. On the one hand, we can obtain a bare or galvanized steel sheet with a TRIP structure by conducting heat treatment of cold-rolled steel sheets on hot-dip galvanizing lines or continuous annealing lines. This process involves annealing the sheets at temperatures covering the two-phase austenitic-ferritic range (780–880 °C), then rapid cooling and isothermal withstanding at bainitic transformation temperatures (350–500 °C) and cooling to ambient temperature. The other method involves carrying out a controlled cooling process after the hot-rolling process. The first stage of the process consists of keeping the sheets after the hot-rolling process on a roller table for a short time in order to have a slow cooling process, which results in an increase in the carbon content in the austenite. Accelerated cooling to the bainitic transformation temperature range is then carried out to stabilize the retained austenite and enrich it with carbon.

The practically described methods of obtaining steel wires and wire rod with TRIP effect are missing in the available literature. Since 2005, a team of scientists from the Częstochowa University of Technology has been the first in the world to undertake research on using TRIP steel for wires and wire products. Basic research carried out under two research projects made it possible to solve the assumed research problems and resulted in many publications [24,25,26,27,28,29]. As part of another research project entitled "Development of an innovative technology and launching the production of steel wire rod with a carbon content of 0.1–0.4% C with a TRIP effect and implementation of the technology of drawing new generation of steel TRIP wires for products manufactured in the drawing industry and metal products", the technology of obtaining wire rods with TRIP structures was developed. The completed project concerned obtaining a wire rod over 5.50 mm diameter. The structure of the TRIP in the wire rod was obtained as a result of regulated cooling process on the Stelmor line. This project imposed the following limitations: in order to obtain a TRIP-type structure in a wire rod, the process of controlled cooling was carried out directly from the temperature of the end of rolling; a dedicated two-stage heat treatment of the TRIP type could not be carried out, which had a direct impact on the content in steel, the most important element of the structure from the viewpoint of the TRIP effect, which is mechanically stable retained austenite. The minimum diameter of the wire rod equal to 5.50 mm was another material limitation. The TRIP wire rod obtained on the Stelmor regulated cooling line, containing a certain, specific amount of retained austenite, is subject to subsequent manufacturing operations in the technological process. The process of deformation by drawing causes the transformation of retained austenite into martensite and leads to its exhaustion in the structure. Accordingly, the final fasteners with preserved retained austenite in the structure cannot be obtained from a wire rod over 5.50 mm diameter.

Hence, a device had to be built for carrying out a two-stage heat treatment of the TRIP-type for wires up to 5.50 mm diameter, to obtain a semi-finished product for the production of fasteners with a large amount of mechanically stable retained austenite (≥60× % C) in steel preserved in the structure, which will be used in subsequent technological operations. The authors aimed to develop two kinds of technology: technology for conducting two-stage heat treatment of TRIP wires up to 5.50 mm diameter for obtaining a TRIP structure. Thanks to this technology, wires with the assumed amount of retained austenite in the structure could be obtained with increased strength properties, constituting a semi-finished product for the production of KPS-6 fasteners used in the construction industry.

Another goal of the authors was to develop technology for the production of steel fasteners with a TRIP effect with increased properties placed on products in the 8.8 grade without conducting heat treatment by hardening and tempering. The results of the research on the development of technology for the production of fasteners will be the subject of another study.

Properly selected chemical composition of steel is an indispensable condition for producing steel products with the TRIP effect. The carbon contents should not be too high, because carbon can be a significant influence on retained austenite stabilization at ambient temperatures. It can cause the transformation of retained austenite into martensite during cold plastic working processes. Adding proper alloying elements such as silicon (Si) and manganese (Mn) also play a very important role in TRIP steels [30,31,32]. Silicon is an element that prevents, or at least delays, the carbide release process during the soaking of steel within the bainitic transformation range, and delays the transformation of retained austenite into martensite. Manganese, like silicon, affects retained austenite stabilization and delays the pearlite forming process. Since the authors intended to develop a technology for obtaining TRIP wire of steel grades, which are mass-produced steels and which do not need to be produced at dedicated heats, two grades of steel with an increased content of silicon and manganese were selected for research in relation to other grades of low-carbon, low-alloy steels commonly used.

Tests of obtaining wires involved the use of a demonstration line to carry out the two-stage process of the TRIP type, which was constructed and developed in industrial conditions, at Metalurgia S.A. Radomsko. According to the results of the research, a semi-finished product could be obtained in the form of TRIP wires with a sufficiently high content of mechanically stable retained austenite, characterised by an increased level of mechanical properties while maintaining adequately high plastic properties.

## 2. Materials and Methods

The chemical compositions of the tested steels are presented in Table 1.

**Figure 1:**
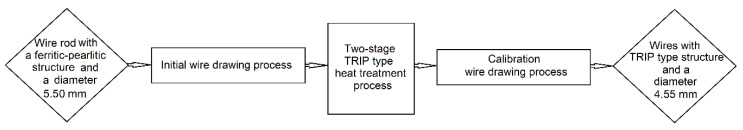
Schematic diagram of research.

The tests included carrying out an initial drawing process of a wire rod with a ferritic–perlitic structure and a diameter of 5.50 mm on a wire with semi-finished product diameters of 5.00 and 4.80 mm, which was then subjected to a two-stage heat treatment process of TRIP types. Subsequently, the heat-treated wires were subjected to a calibration drawing process with a diameter of 4.55 mm in order to prepare the semi-finished product for the production of fasteners in the form of KPS-6 screws used in the construction industry. The test diagram is shown in Figure 1.

The process of drawing a 5.50 mm TRIP steel wire rod on semi-finished product wires with diameters of 5.00 and 4.80 mm was carried out in industrial conditions on the BS700 DRAWING machine. In the process, cone pullers made of sintered carbides with a drawing angle of 2α = 12° were used. The drawing process was carried out at a drawing speed of 6.0 m/s. Table 2 shows the diagram of the single and total crushes used.

Subsequently, wires with a ferritic–perlitic structure and semi-finished product diameters of 5.00 and 4.80 mm were subjected to a two-stage heat-treatment process in order to obtain a TRIP-type structure. For steel in grade S355J2, two diameters of semi-finished product wires were analyzed: 4.80 mm (variant No. 3) and 5.00 mm (variant No. 2), while for steel in grade G4Si1, tests were carried out for a diameter of 5.00 mm (variant No. 1).

Tests of obtaining wires involved the use of a demonstration line to carry out the two-stage heat-treatment process of the TRIP type, which was constructed and developed in industrial conditions, at Metalurgia S.A. Radomsko. The line consisted of:

(a) A passive disc unwinder with a friction brake with adjustable clamping force of the rotating disc, which reduces excessive wire overhang during the unwinding process;
(b) An arrangement of drawing rollers with individual drive and a set of stress-relieving rollers mounted in two planes, which reduce the intrinsic stresses of the wire;
(c) An induction coil capable of achieving the required temperature (850 °C) in less than 5 s;
(d) A high-temperature pass-through furnace No. I (850 °C);
(e) A through cooler with adjustable compressed air blow to achieve the required cooling speed;
(f) A low-temperature pass-through furnace II (440 °C);
(g) A winder with a smooth adjustment of the rotational speed of the drum with a diameter of 1000 mm.

**Figure 2:**
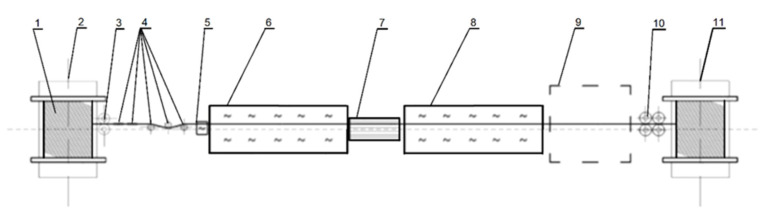
Diagram of a technological line for two-stage heat treatment process for obtaining wires with TRIP structure (1—material; 2—decoiler; 3—drawing rollers; 4—straightening rollers; 5—heating device; 6—high-temperature continuous furnace; 7—continuous cooler; 8—continuous low-temperature furnace; 9—free cooling area; 10—drawing rollers; 11—drum winder).

The diagram of the process line is shown in Figure 2.

As part of the research, technological tests were carried out using a demonstration line to carry out two-stage heat treatment of the TRIP type in order to determine the production readiness of each of the heating zones, ensuring the required temperatures and the appropriate cooling speed were achieved; that is, the parameters that determine the TRIP structure being obtained. Table 3 summarizes the results of the measurements made.

Induction heating tests of the previously prepared wire were carried out with a throughput speed of 6.7 and 8.9 cm/s for diameters of 4.8 and 5.0 mm. The conducted heating tests confirmed the achievement of the set temperature (850 °C) in the assumed time of less than 5 s. Tests of heating in the two-phase range by retaining elements in the working area of the furnace No. 1 were also carried out. Tests of the cooling zone efficiency from the heating temperature to the bainitic transformation temperature were carried out. The obtained results confirmed the effectiveness of the accelerated cooling zone in the range of 30–50 ºC/s. As part of the assessment of the efficiency of the demonstration line, tests were also carried out to confirm the stability of the temperature in the range of bainitic transformation temperatures for the working area of furnace II.

After reaching the production readiness of the technological line for two-stage heat treatment of the TRIP type, tests were carried out for two steel grades: for steel in the S355J2 grade, two diameters of semi-finished product wires were analyzed: 4.80 mm (variant No. 1) and 5.00 mm (variant No. 2), while for steel in the G4Si1 grade, tests were carried out for a diameter of 5.00 mm (variant No. 3).

On the basis of the chemical composition of the tested steels, the temperature ranges in which phase transformations take place were determined [30] using CTPc graphs. This allowed us to determine the ranges of the thermal treatment process parameters of the TRIP type during cooling.

Table 4 presents the parameters of heat treatment carried out in order to obtain a TRIP type structure in heat-treated wires.

The subsequent research stage included carrying out the calibration drawing process for wires with a TRIP structure and diameters of 5.00 and 4.80 mm for the final diameter of 4.55 mm, which is the output dimension for the semi-finished product: wires with a TRIP structure used to produce fasteners in the form of KPS-6 screws used in the construction industry. The calibration drawing process was carried out on the JP600 drum drawing machine. In the process, cone pullers made of sintered carbides with a drawing angle of 2α = 12° were used. The drawing process was carried out at a drawing speed of 1.6 m/s. The wires were pulled in one calibration drawn. The wires with a diameter of 5.00 mm were pulled to the final dimension of 4.55 mm with a single crush equal to 17%. On the other hand, wires with a diameter of 4.80 mm were pulled to the final dimension of 4.55 mm, with a single crimp equal to 9.9%.

The aim of the research carried out in the thesis was to obtain a semi-finished product in the form of TRIP wires constituting the starting material for the production of fasteners in the form of KPS-6 screws used in the construction industry. These wires should have an appropriate content in the structure of mechanically stable retained austenite, which will be used in subsequent technological stages regarding the production of fasteners and should have increased values of strength properties with high plastic properties in relation to wires with a ferritic and perlitic structure.

Respectively, the wires after each technological operation were placed under tests of mechanical properties. Tests of mechanical properties of drawn and heat-treated wires, after each stage of the technological process, were carried out in accordance with PN-EN ISO 6892-1:2010 on the Zwick/Z100 strength machine.

Metallographic tests were also carried out. These tests consisted of the analysis of the structure of heat-treated wires using the JEOL 2100 Plus electron microscope, confirming the presence of retained austenite in the structure of heat-treated wires.

The amount of retained austenite in the structure of heat-treated wires was determined using three methods. In order to determine the phase composition, a metallographic analysis of the obtained structures was carried out with optical microscopy with an Axiovert 25 optical microscope and the Veeco Multimode V atomic force microscope, and structural components were identified in heat-treated samples according to various variants.

The amount of retained austenite in the wires was also determined with X-ray diffraction using the Bruker D8 Advance X-ray diffractometer.

## 3. Results and Discussion

### 3.1. Qualitative Metallographic Analysis of Heat-Treated Wires with JEOL 2100 Plus Electron Microscope

Retained austenite in the form of "free" individual grains occurred in the analyzed steels in a small amount. Retained austenite may be present in some small volume in the mixture with other phases in the form of so-called martensitic–austenitic islands or martensitic/bainitic–austenitic islands.

Hence, in order to confirm the presence of retained austenite in the structure of heat-treated wires, qualitative tests of the sample microstructure were carried out with a JEOL 2100 Plus transmission electron microscope. Samples in the form of thin films obtained by electrolytic etching in a two-stream Struers Tenupol VII shaper were tested.

**Figure 3:**
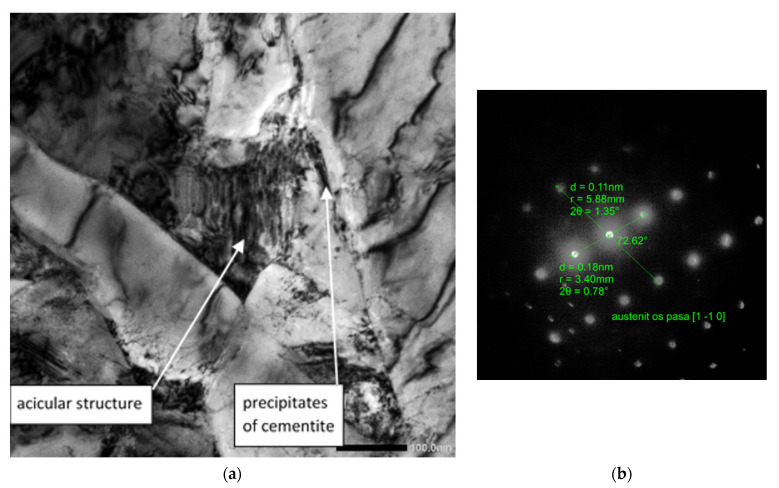
An image of the microstructure for variant 1; (**a**). Microstructure with visible bainitic and martensitic island (B + M) of very fine coniferous structure, image in bright field magnification 15 kx; (**b**). Electron diffraction from retained austenite in B + M island. The axis of the austenite belt [1 $\bar{1}\;0]$.

**Figure 4:**
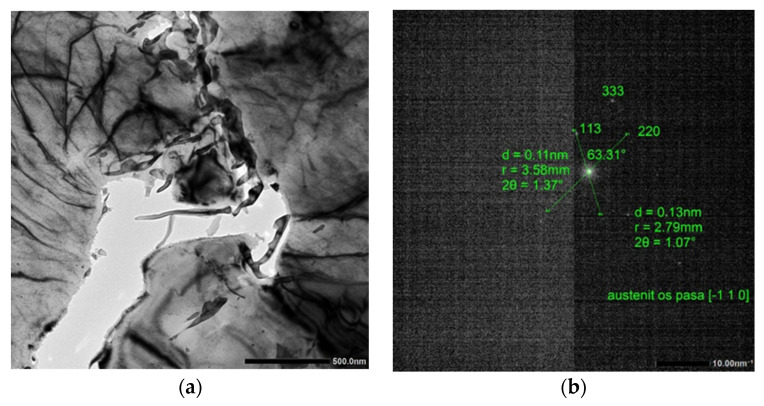
Image of the microstructure for variant 2; (**a**). Ferritic microstructure with visible B + M island, image in the bright field magnification 15 kx; (**b**). Electron diffraction from retained austenite in a martensitic island. Austenite belt axis [1 $\bar{1}\;0]$.

**Figure 5:**
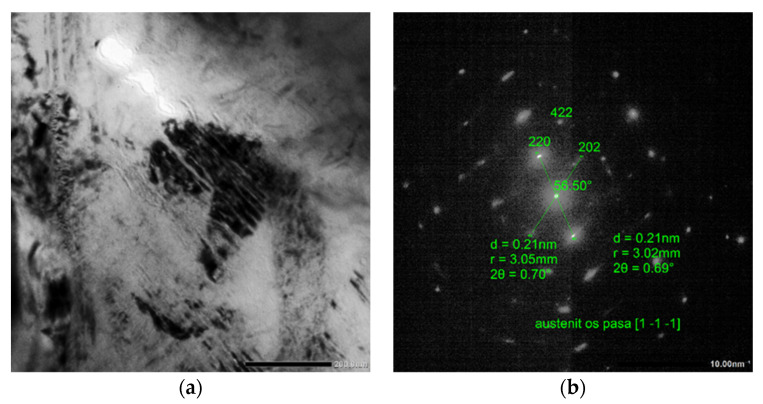
An image of the microstructure for variant 3; (**a**). Microstructure of grain with a bainitic-martensitic (B + M) area visible inside with a very fine coniferous structure, image in a bright field magnification of 40 kx; (**b**). Electron diffraction from retained austenite in B + M island. The axis of the austenite belt [1 $\bar{1}\bar{1}]$.

The test results are presented in Figure 3, Figure 4 and Figure 5.

In Figure 3, we observe a ferritic–bainitic microstructure with visible areas of ferrite. Large grains, in the middle, show the present martensitic island with a coniferous structure; between the needles of the martensitic phase, the retained austenite is clamped. High-density dislocation ferrite was present in the structure. At the boundary of the ferrite grains, we observe the separation of cementite.

In Figure 4, we observe a ferritic and bainitic microstructure with visible areas of ferrite (large grain); the dark part in the middle above the hole is a bainitic and martensitic island with a coniferous structure. Retained austenite is clamped between the martensitic phase needles. High-density dislocation ferrite, partially polygonized, is present in the structure. At the boundary of the ferrite grains running upwards, we observe the separation of cementite.

In Figure 5, we observe the ferrite grains partially transformed into a coniferous structure (bainitic –martensitic, visible as a dark part with a high density of dislocation). Inside the transformed area there is retained austenite after the boundary of the slats.

From the presented test results, it should be concluded that the two-stage heat treatment process of the TRIP type, carried out in accordance with the parameters set out in Table 4, allowed us to obtain a structure in which we identify the presence of retained austenite: a structural component directly responsible for obtaining a favourable relationship between mechanical properties and plasticity.

### 3.2. Quantitative Analysis of the Share of Retained Austenite in the Structure of Wires Subjected to Two-Stage Heat Treatment Type TRIP

The amount of retained austenite in the structure of heat-treated wires was determined using three methods.

**Figure 6:**
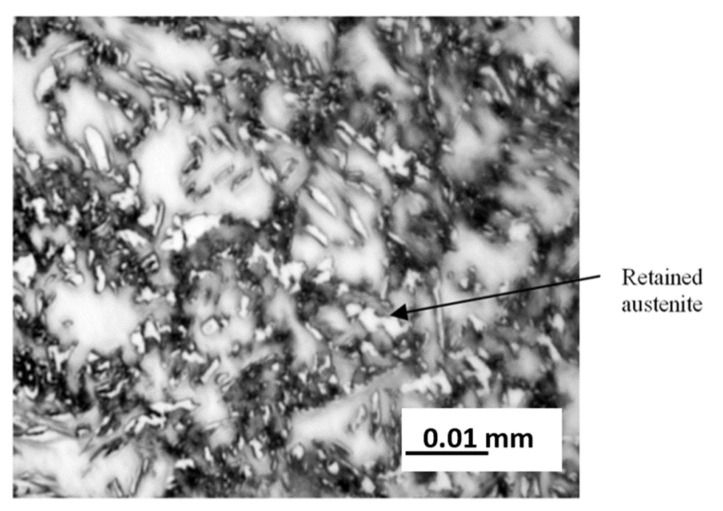
Microstructure of multiphase steel, variant no 2.

The one method was to determine the phase composition of materials by using optical microscopy with Axiovert 25 optical microscope. In order to extract retained austenite in the structure, the digestion of heat-treated samples was carried out with 2.5% nital solution, and next with 10% sodium metabisulfite solution [33]. Thanks to this method, retained austenite can be extracted, because this reagent etches it to a white colour. Figure 6 shows an image of the microstructure for the heat-treated sample according to variant No. 2.

The volume share of retained austenite in the structure of heat-treated wires was calculated in accordance with three metallographic methods used for quantitative analysis: the point method, the incision method and the MET-ILO computer program, while in Table 5, column 2, the averaged results for all three methods were included.

The other method used in the research to determine the amount of retained austenite in the structure of heat-treated wires was the research carried out with the atomic force microscope.

**Figure 7:**
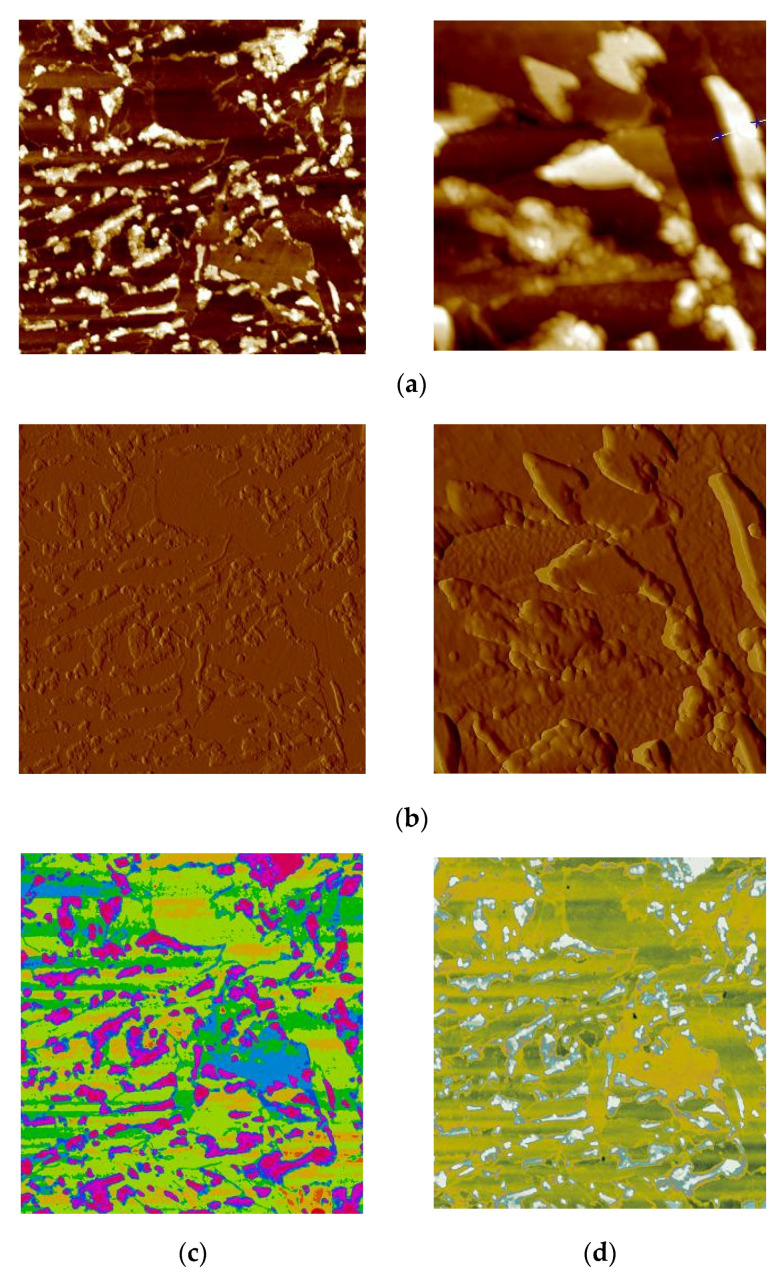

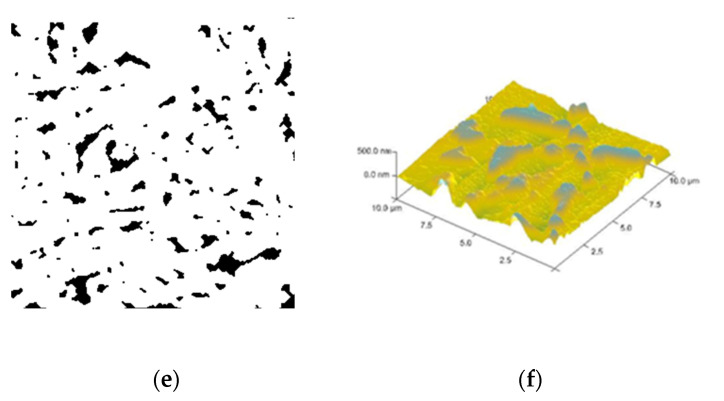
An image of the microstructure of samples heat-treated according to variant no. 1: (**a**) topography of the tested surface in semi-contact mode; (**b**) surface mapping with amplitude contrast; (**c**) surface topography in a 10-degree colour scale; (**d**) comparative control image with a limited continuous colour palette; (**e**) binary image after digitization of the austenitic phase; (**f**) 3D view of the sample surface.

A fragment for inclusion was taken from each sample. Then, polishing was carried out on diamond paste. To avoid oxidation of the surface after polishing, the samples were immediately etched in nital (2% nitric acid; 98% ethanol) for 15 to 17 s. This reagent digests morphological components at different speeds; ferrite is etched the fastest, followed by bainite and martensite, while austenite is the slowest. Carbides remain almost non-etched. With a scanning microscope with a probe, after etching, the individual phases may be inferred based on their height. The excretions were visible as the highest points due to their very weak reaction with the reagent. Hence, individual structural components may be distinguished, and their contributions may be quantified. To this end, areas with an area of at least 2500 μm^2^ were scanned with a Veeco Multimode V atomic force microscope. After obtaining an image in the semi-contact mode showing topographic (high) differences, it was processed in order to quantify the elements at a height. They were quantified into 10 classes marked with colours. In the subsequent step, the colour palette was edited to increase contrast and eliminate the divisions. The editing with the use of a colour palette consisted of its selection in such a way as to clearly separate the separations (of high height) and ferritic areas—the most etched (the lowest). After this operation, the colour mask for the analysis was downloaded with Corel Photo Paint X7. The mask saved in this way was developed by Image Analysis Pro in order to eliminate artefacts: single pixels, holes, etc. After preparing the image, the surface share of retained austenite in the structure of the tested samples was determined. In Figure 7, an image of the microstructure of heat-treated samples according to variant No. 1 is shown.

The amount of retained austenite in the structure of heat-treated wires was also determined with X-ray diffraction.

**Figure 8:**
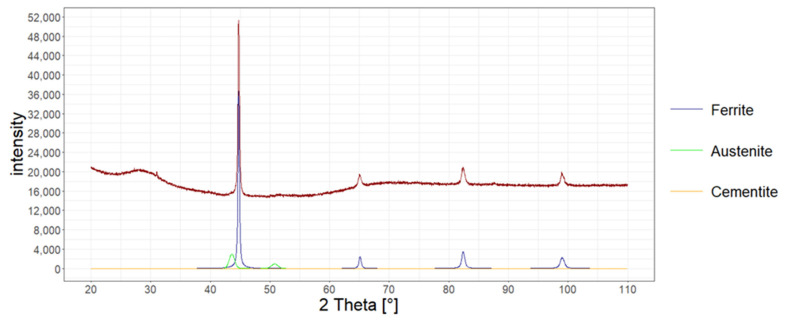
Phase graph of the X-ray diffraction of the tested heat-treated steel according to variant No. 1.

**Figure 9:**
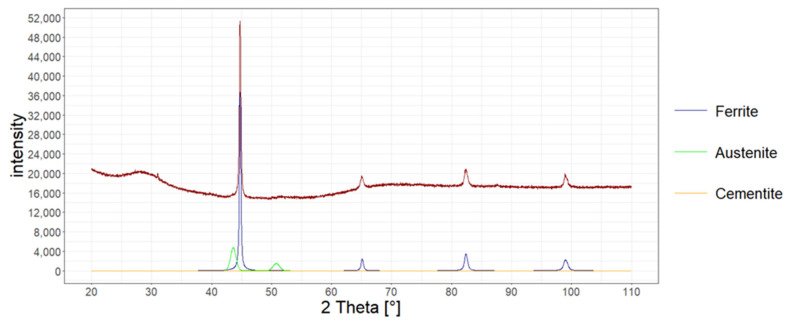
Phase graph of the X-ray diffraction of the tested heat-treated steel according to variant No. 2.

**Figure 10:**
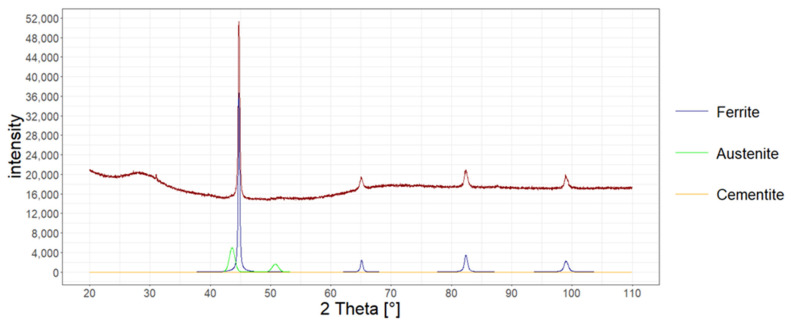
Phase graph of the X-ray diffraction of the tested heat-treated steel according to variant No. 3.

The measurement was performed in accordance with the Bragg–Brentano method on the Bruker D8 Advance X-ray diffractometer for the account ranging from 20 to 110 degrees. Copper lamp and CuKα radiation were applied. The measurement was carried out every 0.02 degrees. The analysis was carried out in accordance with the Rietveld method, assuming the existence of two phases of ferrite and austenite. In Figure 8, Figure 9 and Figure 10, X-ray diffraction phase graphs for all heat treatment variants are presented.

Table 5 presents the results of the research on the amount of retained austenite for heat-treated wires according to various variants using the quantitative method, microstructure analysis using the atomic force microscope, and X-ray diffraction.

Tests of the amount of retained austenite in wires with different heat-treated diameters with different process parameters allowed us to determine the effectiveness of individual zones of the demonstration line, especially the accelerated cooling zone.

The study aimed to obtain wires with the maximum possible content of mechanically stable retained austenite in the structure, which will be used in subsequent stages of manufacturing fasteners. According to the literature data, a two-stage heat treatment TRIP in laboratory conditions may yield up to 10% of retained austenite for each 0.1% C with a properly conducted process.

The authors’ assumption was to obtain, in wires, a multiphase TRIP-type structure with retained austenite in the amount of ≥60×% C in steel, because the process was carried out in industrial conditions featuring a high variability of factors influencing the achievement of the assumed structure.

For S355J2-grade steel, an average of over 11% of retained austenite was obtained in the structure of wires with a diameter of 4.80 mm and about 11% for wires with a diameter of 5.00 mm. Hence, the adopted preliminary assumptions have been met, because the steel in the S355J2 grade contains 0.18% C.

For steel of G4Si1 grade, an average of about 7% of retained austenite was obtained in the structure of wires with a diameter of 5.00 mm. Hence, the adopted preliminary assumptions have been met, because the steel in the G4Si1 grade contains 0.08% C.

### 3.3. Analysis of the Results of Mechanical Properties

The mechanical properties of the wires were examined at several stages of wire production: after the pre-drawing process before the two-stage heat treatment process of the TRIP type, after the two-stage heat treatment process of the TRIP type, and after the calibration drawing process. The test results are shown in Table 6 and Table 7.

The analysis of the results of the tests of mechanical properties of the semi-finished product—that is, TRIP wires—allows us to state that as a result of the heat treatment and the process of initial reinforcement of wires in the calibration drawing process, a semi-finished product with an increased level of mechanical properties was obtained in relation to wires with a typical ferritic and perlitic structure. The values of tensile strength were about 100 MPa higher. However, an increase in the plasticity of the material was found, which was caused by the presence of retained austenite in the structure of TRIP wires. The ratio of R_e_ to R_m_ was sufficiently low; that is, the semi-finished product in the form of TRIP wires, has a sufficiently large plasticity reserve, which can be used to properly shape the product—mounting screws—in the process of fastening the head and rolling the thread. The retained austenite identified in the structure of the tested wires is absolutely responsible for the situation; it clearly affects the increase in plasticity in relation to the starting material, resulting in a two-fold increase in the value of A_50_, which was recorded during the wire stretching test after two-stage heat treatment. The R_e_ value remains at a similar level as for the starting material, but it clearly increases R_m_, which is related to the change in the morphology of the structure and the increase in the dispersion of structural components of the tested wires. The execution of the calibration sequence inevitably leads to the occurrence of the transformation of retained austenite into martensite in the structure; the effect of this process becomes noticeable in the results of both Rm and Retests. Both strength parameters clearly increase the values; while in the case of Rm this increase was about 100–150 MPa, the increase in R_e_ ranged from 200 to 280 MPa.

The conducted process, consisting of two-stage heat treatment and a calibration sequence, leads to an increase in strength properties in selected steels, but what is particularly interesting is that it maintains the recorded elongation of A_50_ at a similar level, whereby joint products may be shaped by thread rolling and shaping the screw head.

Both of these operations will result in a further increase in mechanical properties and a product showing the level of mechanical properties specified for fasteners in property class 8.8 without thermal treatment by hardening and tempering.

## 4. Conclusions

1. The applied parameters of the two-stage TRIP heat treatment process on the demonstration line for the analyzed steel grades allowed us to obtain wires with up to 5.50 mm diameters with a volume fraction of retained austenite in the structure, meeting the assumed requirements: ≥60×%C in steel.
2. Wires after TRIP-type heat treatment process have higher values of tensile strength in comparison to unheated wires with ferritic–pearlitic structure.
3. The conducted two-stage TRIP-type heat treatment process and the subsequent calibration drawing process allowed us to obtain the material with an appropriate plasticity reserve (R_e_/R_m_ at the level of 0.84–0.87); that is, raw material for the production of screws.
4. The successive stages of shaping the screw in the plastic deformation processes will result in the transformation of the retained austenite into martensite, which will increase the mechanical properties and obtaining a fastener in property class 8.8 without conducting heat treatment on the finished product.
5. The techniques of determining the share of retained austenite used in the presented work, both stereological and X-ray structural methods, offer high consistency in the obtained values and can be used interchangeably.
6. The applied parameters of the two-stage TRIP type heat treatment process allowed us to obtain a material with 11% of the volume fraction of retained austenite in the structure for steel grade S355J2 and 7% for steel grade G4Si1.
7. A technological line developed in industrial conditions for conducting a two-stage TRIP heat treatment process for wires with diameters below 5.50 mm gives the possibility of producing semi-finished products, such as wires for ropes used in the construction of protective barriers on motorways.

## Figures and Tables

**Table 1:** Chemical composition of tested steels.

Grade of Steel	C %	Mn %	Si %	P %	S %	Cr %
G4Si1	0.08	1.44	0.84	0.008	0.019	0.03
S355J2	0.18	1.39	0.22	0.011	0.008	0.03

**Table 2:** Diagram of the single (G_p_) and total reduction (G_c_) used in the drawing process of a wire rod with a ferritic–perlitic structure and a diameter of 5.50 mm for a wire with semi-finished product diameters of 5.00 and 4.80 mm.

Drawn No.	Φ mm	G_p_, %	G_c_, %
0	5.50	-	-
1	5.20	10.61	10.61
2	5.00	7.54	17.35
3	4.80	7.84	23.83

**Table 3:** Thermally treated wire temperature measurement results for wire speed v = 6.7 cm/s.

Wire Diameter	Ambient Temp.	Temp. after the Inductor Coil	Temp. T1	Temp. at the Exit of Furnace No.1	Temp. after the Cooler	Temp. T2	Temp. at the Exit of Furnace No.2
mm	°C	°C	°C	°C	°C	°C	°C
4.8	15	862	860	860	431	430	430
4.8	15	858	850	851	417	420	421
4.8	17	873	870	872	455	450	453
5.0	16	824	820	821	416	440	440
5.0	17	847	840	844	457	460	461

**Table 4:** TRIP-type heat treatment process parameter range.

Variant		Wire Diameter	Temp. after the Inductor Coil	Temp. T1	Wire Speed, v	Temp. after the Cooler	Temp. T2	Grade of Steel
		mm	°C	°C	cm/s	°C	°C	
1	assumed parameters	4.8	770	750	6	350	350	S355J2
	real parameters	4.8	772	750	6	349	350	
2	assumed parameters	5.0	770	750	6	350	350	
	real parameters	5.0	771	750	6	348	350	
3	assumed parameters	5.0	790	790	6	370	370	G4Si1
	real parameters	5.0	791	790	6	365	370	

**Table 5:** Amount of retained austenite obtained in heat-treated wires.

Variant No.	Quantity of Retained Austenite Determined in Accordance with Quantitative Methods %	Amount of Retained Austenite Determined in Accordance with Atomic Force Microscope %	The Amount of Retained Austenite Determined in Accordance with X-ray Diffraction %
1	11.2	10.4	11.9
2	10.9	10.6	11.4
3	6.9	6.60	7.06

**Table 6:** Mechanical properties of wires before and after the two-stage heat treatment process, TRIP-type.

Variant No.	Wire Diameter mm	R_m_, MPa	R_e,_ MPa	R_e_/R_m_	A_50_, %
Pre-treatment wires prior to the two-stage TRIP process					
1	4.8	550	423	0.76	15.36
2	5.0	530	415	0.78	13.24
3	5.0	520	402	0.77	14.23
Wire following a two-stage TRIP heat treatment process					
1	4.8	623	402	0.64	29.66
2	5.0	623	358	0.57	30.27
3	5.0	583	386	0.66	34.36

**Table 7:** Mechanical properties of wires after the calibration drawing process.

Variant No.	Steel Grade	Initial Diameter, mm	Final Diameter, mm	R_m_, MPa	R_e,_ MPa	R_e_/R_m_	A_50_, %
1	S355J2	4.80	4.55	720	605	0.84	15.2
2	S355J2	5.00	4.55	750	642	0.86	18.2
3	G4Si1	5.00	4.55	730	635	0.87	14.3

## Data Availability

Data sharing is not applicable to this article.

## References

[1] Grajcar A., Kozłowska A., Radwański K., Skowronek A. (2019). Quantitative Analysis of Microstructure Evolution in Hot-Rolled Multiphase Steel Subjected to Interrupted Tensile Test. Metals.

[2] Gronostajski Z., Niechajowicz A., Kuziak R., Krawczyk J., Polak S. (2017). The Effect of the Strain Rate on the Stress-Strain Curve and Microstructure of AHSS. J. Mater. Process. Technol..

[3] Vercruysse F., Cerda F.M.C., Verleysen P., Petrov R.H. (2020). Behavior of Ultrafast Annealed Advanced High Strength Steels Under Static and Dynamic Conditions. Mater. Sci. Eng. A.

[4] Raabe D., Sun B., da Silva A.K., Gault B., Yen H.W., Sedighiani K., Sukumar P.H., Filho I.R.S., Katnagallu S., Jagle E. (2020). Current Challenges and Opportunities in Mi-crostructure-Related Properties of Advanced High- Strength Steels. Metall. Mater. Trans. A.

[5] Bordone M., Monsalve A., Ipina J.P. (2022). Fracture toughness of High-Manganese steels with TWIP/TRIP effects. Eng. Fract. Mech..

[6] De Cooman B.C., Estrin Y., Kim S.K. (2018). Twinning-induced plasticity (TWIP) steels. Acta Mater..

[7] Mitter W. (1987). Umwandlungsplastizität und ihre Berücksichtigung bei der Berechnung von Eigenspannungen, Metallkundliche- Technische Reihe 7.

[8] Zackay V.F., Parker E.R., Fahr D., Bush R. (1967). The enhancement of ductility in high-strength steels. Trans. Am. Soc. Met..

[9] Berrahmoune M.R., Berveiller S., Inal K., Moulin A., Patoor E. (2004). Analysis of the martensitic transformation at various scales in TRIP steel. Mater. Sci. Eng. A.

[10] Grajcar A., Kilarski A., Kozłowska A., Radwański K. (2019). Microstructure Evolution and Mechanical Stability of Retained Austenite in Thermomechanically Processed Medium-Mn Steel. Materials.

[11] Eres-Castellanos A., Caballero F.G., Garcia-Mateo C. (2020). Stress or Strain Induced Martensitic and Bainitic transformations during ausforming processes. Acta Mater..

[12] Frolova A.V., Stolyarov V.V., Kumar J.V.T., Sudha J. (2021). Features of TRIP steel deformation at low and moderate temperatures. Mater. Sci. Eng..

[13] Qiu J., Zhang M., Liu X., Zhang X., Tan Z. (2020). Characterization of Retained Austenite in a Low Carbon High Strength Mn-Si-Cr Steel. Mater. Sci. Eng. A.

[14] Long X., Zhao G., Zhang F., Xu S., Yang Z., Du G., Branco R. (2020). Evolution of Tensile Properties with Transformation Temperature in Medium-Carbon Carbidefree Bainitic steel. Mater. Sci. Eng. A.

[15] Vercruysse F., Celada-Casero C., Linke B.M., Verleysen P., Petrov R.H. (2020). Temperature Dependence of the Static and Dynamic Behaviour in a Quenching and Partitioning Processed Low-Si Steel. Metals.

[16] Lloyd J.T., Magagnosc D.J., Meredith C.S., Limmer K.R., Field D.M. (2022). Improved dynamic strength of TRIP steel via pre-straining. Scr. Mater..

[17] Prosvirnin D.V., Kolmakov A.G., Larionov M.D., Pivovarchik S.V. (2021). Peculiarities of deformation of thin-sheet TRIP steel under static and fatigue loading. J. Phys. Conf. Ser..

[18] Kozłowska A., Grajcar A., Radwański K., Opara J., Matus K., Nuckowski P.M. (2022). Microstructure and temperature-dependent mechanical behavior of hot-rolled TRIP-assisted microalloyed steel. Mater. Charact..

[19] Kozłowska A., Grajcar A. (2020). Effect of Elevated Deformation Temperatures on Microstructural and Tensile Behavior of Si-Al Alloyed TRIP-Aided Steel. Materials.

[20] Girault E., Mertes A., Jacques P., Houbaert Y., Verlinden B., Humbeeck J. (2001). Comparison of the effects of silicon and aluminium on the tensile behavior of multiphase TRIP-assisted steels. Scr. Mater..

[21] Covarrubias O., Guerrero M.P., Colás R., Petrov R., Kestens L., Houbaert Y. Transformation behaviour of Si and Mn bearing low carbon steels. In Proceeding of the TRIP—International Conference on TRIP-Aided High Strength Ferrous Alloys.

[22] Oja O., Saastamoinen A., Patnamsetty M., Honkanen M., Peura P., Järvenpää M. (2019). Microstructure and Mechanical Properties of Nb and V Microalloyed TRIP-Assisted Steels. Metals.

[23] Salinas A., Artigas A., Perez-Ipiña J., Castro-Cerda F., Garza-Montes-de-Oca N., Colás R., Petrov R., Monsalve A. (2018). Effects of Heat Treatment on Morphology, Texture, and Mechanical Properties of a MnSiAl Multiphase Steel with TRIP Behavior. Metals.

[24] Wiewiórowska S., Muskalski Z. (2015). The influence of partial single reduction on mechanical properties wires made from TRIP steel with 0.43%C. Metalurgija.

[25] Suliga M., Muskalski Z. (2009). Wiewiórowska, The influence of drawing speed on fatigue strength TRIP steel wires. Arch. Civ. Mech. Eng..

[26] Siemiński M., Wiewiórowska S., Muskalski Z. (2016). Examination of the Effect of Variation in Stress Magnitude on the Amount of Transformed Retained Austenite in the Structure of TRIP Steel Wires. Key Eng. Mater..

[27] Wiewiórowska S., Muskalski Z., Siemiński M. (2016). The analysis of “hot” drawing process of TRIP steel wires at different initial temperatures. Arch. Metall. Mater..

[28] Wiewiórowska S., Muskalski Z., Michalczyk J. (2019). The Influence of Hot Dip Galvanizing Process on Trip Steel Wire Structure and Properties. Arch. Metall. Mater..

[29] Kucharska M., Wiewiórowska S., Michalczyk J., Gontarz A. (2020). The Influence of the Drawing Process on the Mechanical Properties of TRIP Steel Wires with 0.4% C Content. Materials.

[30] Tomita Y., Morioka K. (1997). Effect of microstructure on Transformation-Induced-Plasticity of silicon-containing low-alloy steel. Mater. Charact..

[31] Ostash O.P., Kulyk V.V., Poznyakov V.D., Gaivorons’kyi О.А., Vira V.V. (2019). Influence of the Modes of Heat Treatment on the Strength and Cyclic Crack-Growth Resistance of 65G Steel. Mater. Sci..

[32] Xu B., Chen P., Li Z., Wu D., Wang G., Guo J., Liu R., Misra R.D.K., Yi H. (2021). The Significance of Optimizing Mn-Content in Tuning the Microstructure and Mechanical Properties of δ-TRIP Steels. Metals.

[33] ASM Handbook Committee (2002). Properties and Selection: Irons Steels and High Performance Alloys.

